# Current applications and future directions in natural language processing for news media and mental health

**DOI:** 10.1038/s41598-025-18413-z

**Published:** 2025-09-15

**Authors:** Jannis Köckritz, Bahar İlgen, Caroline Cohrdes, Georges Hattab

**Affiliations:** 1 Center for Artificial Intelligence in Public Health Research (ZKI-PH), Robert Koch Institute, Berlin, 13353 Germany; 2 Department of Mathematics and Computer Science, Freie Universität Berlin, Berlin, 14195 Germany; 3 Department of Epidemiology and Health Monitoring, Mental Health Research Unit, Robert Koch Institute, Berlin, 13353 Germany

**Keywords:** NLP, News media, Mental health, Computer science, Public health, Psychiatric disorders

## Abstract

Mental health discourse has gained prominence in public media, significantly influencing societal perceptions. This study explores the application of Natural Language Processing (NLP) techniques to analyze the representation of mental health in news media texts. The complex interplay between media representation and public understanding of mental health requires advanced analytical tools. NLP offers promising avenues for unpacking these narratives but faces challenges in capturing the nuances of mental health discourse. We employ a scoping review to examine several NLP applications, including sentiment analysis, topic modeling, and bias detection. Our study compares news media to social media, highlighting the unique linguistic challenges of formal journalistic language. The analysis reveals significant limitations of current NLP techniques when applied to mental health news coverage. We uncover significant biases and accuracy issues in sentiment analysis of mental health content across different media platforms. Our findings underscore the need for specialized NLP techniques in mental health news analysis. We propose ten recommendations for tailored NLP approaches that provide critical insights for researchers, policymakers, and media professionals. This work aims to improve mental health communication strategies and promote more nuanced, effective public discourse and media coverage.

## Introduction

Mental health has recently received significant media attention, bringing it into the public eye. A critical aspect of this visibility is how mental illness is portrayed in the media. Numerous publications have shown that mental illness is frequently depicted in a dramatic and inaccurate light, emphasizing danger, criminality, and unpredictability^1–4^. Such distorted portrayals shape public perceptions and perpetuate stigma and discrimination against people with mental illness. These negative portrayals can lower self-esteem^5^ and discourage help-seeking behavior. They can also reduce medication adherence and impair overall recovery outcomes^6^. As a result, these effects can delay treatment, increase symptom severity, and worsen the long-term prognosis for those affected by mental illness^7^.

From a public health perspective, addressing media portrayals of mental health is critical to both promoting well-being and achieving health equity. The media is a primary source of information for the public and shapes societal norms and beliefs about mental health. Misrepresentation and stigma in the media can exacerbate existing prejudices, create barriers to effective mental health care, and increase health disparities. This dynamic is particularly harmful for marginalized groups who may already face greater barriers to accessing care. Accurate portrayals can challenge these barriers by providing a more compassionate, inclusive understanding of mental health that supports well-being and helps reduce disparities in mental health outcomes^6^. Balanced and factual portrayals of mental health in the media are essential to normalizing discussions about mental health, promoting early intervention, and encouraging people to seek help. For example, positive portrayals of people recovering from mental illness can reduce stigma and encourage others to seek care, ultimately contributing to a healthier society^8,9^. In this way, the media has the potential to actively promote the mental well-being of the population by reducing fear and misunderstanding of mental illness.

Given the wide reach of the media, the responsibility to present a realistic and balanced picture of mental health is essential. Natural Language Processing (NLP) provides an opportunity to analyze and shape media portrayals, identifying both harmful and constructive narratives that influence public perception. Advocacy groups, such as the *Time to Change* campaign in the United Kingdom^10^, hold the media accountable for promoting stigma and discrimination, while recognizing its potential to combat public prejudice through positive storytelling. NLP can support these efforts by systematically quantifying the extent and nature of stigmatizing narratives in large media datasets. For example, using co-occurrence network analysis, researchers can identify how mental health terms (e.g., “depression,” “schizophrenia”) are frequently associated with negative contexts (e.g., “violence,” “unemployment”), highlighting where stereotypes persist. This technique provides evidence-based insights to inform public awareness campaigns, allowing advocacy groups to target specific harmful narratives. Research suggests that targeted interventions, particularly those involving education and contact with people with mental illness, can be effective in reducing stigma^6,11^.

In addition, media discussions often reflect societal responses to mental health issues, and their analysis can provide insights into population-level mental health trends. For example, NLP techniques such as sentiment analysis, emotion detection, and event extraction can be used to assess media responses to societal events, such as economic crises or health emergencies, and can reveal patterns of anxiety, depression, and well-being at the population level^12,13^. For example, during the COVID-19 pandemic, event extraction could automatically identify key moments (e.g., lockdowns, economic downturns) associated with spikes in anxiety or depression in media discussions, helping researchers identify which events contribute to shifts in public sentiment about mental health^12,13^.

This method also allows public health officials to monitor real-time responses to crises, providing actionable insights for public mental health interventions. Understanding how mental health narratives are constructed and communicated is critical, especially with large-scale media data. NLP techniques offer robust tools for analyzing these narratives and identifying recurring themes, biases, and emotional tones that shape public perceptions. This study explores the potential of NLP techniques to analyze how mental health topics such as depression, anxiety, and well-being are represented in online news media. Specifically, we assess how NLP techniques can detect and interpret complex constructs, which in psychology refer to concepts that describe real-world phenomena^14^.

Our review highlights the precision and depth that advanced NLP techniques, such as transformer-based architectures and contextual embeddings, offer in identifying nuanced expressions of mental health issues in media narratives. Techniques such as topic modeling, emotion detection, and named entity recognition (NER) are used to capture how complex mental health constructs are framed and interpreted in news content. By focusing on how these techniques extract and analyze the subtle language used in mental health discussions^15^, we demonstrate how they contribute to a more informed understanding of the media’s influence on public perceptions of mental health. To improve the accuracy and balance of media portrayals of mental health, we propose ten recommendations for the development of NLP tools specifically tailored to mental health and news media analysis. By advancing NLP techniques in mental health media analysis, we aim to provide practical insights for developers to enable tools that more accurately capture both the stigmatizing and destigmatizing elements of mental health discussions in the news.

**Fig. 1:**
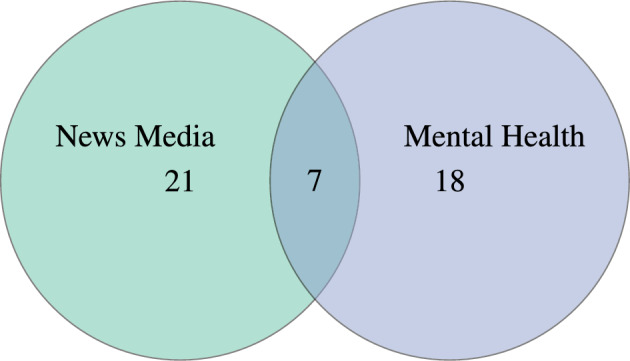
Distribution of the Related Work on News Media, Mental Health, and their intersection. This Venn diagram illustrates the categorization of the included publications into three distinct groups. Of the publications, twenty were solely about news media, fourteen were solely about mental health, and six examined the intersection of NLP for news media and mental health.

This review aims to synthesize and critically assess how natural language processing has been applied to the study of mental health in news media. While research on news media and on mental health has each grown substantially, their intersection remains underexplored. As shown in Fig. 1, most retrieved studies focus on one domain in isolation, with only a small subset addressing both. This imbalance motivates the present review.

## Overview of NLP applications in mental health and news media

In this section, we present our results from the scoping review by considering the grouping of traditional and artificial intelligence-based NLP techniques. We then highlight our findings in the context of each group: traditional content analysis methods, NLP for news media, NLP for mental health, and its portrayal. As NLP has become increasingly important to the study of news media and mental health, our scoping review includes relevant research since 2012.

Advances in the field of NLP have allowed researchers to address misinformation and mental health issues. This is exemplified by the COVID-19 pandemic^16,17^. The COVID-19 pandemic has underscored the urgency of developing effective NLP techniques. The pandemic has led to a surge in mental health issues due to increased stress, anxiety, and social isolation^18,19^. Ultimately, it has accelerated research to address these new challenges and mitigate long-term effects. Similarly, the pandemic has heightened the spread of misinformation, necessitating the development of NLP tools to detect and combat fake news early^20,21^.

This review follows an exploratory, purposive approach rather than a systematic protocol, aiming to surface diverse methodological applications of NLP in the context of mental health and news discourse. We used combinations of the search terms “mental health”, “news media”, and “natural language processing”, selected for their alignment with controlled vocabularies in public health and computer science, as well as their broad coverage across domains. Alternative or narrower terms (e.g., “depression,” “newspapers,” “media framing”) were considered during pilot searches but yielded results that were either overly clinical, limited to social media, or too narrow in scope. We thus retained the broader terms to ensure inclusiveness and conceptual coverage across disciplines.

Sources consulted include Google Scholar, Scopus, Web of Science, arXiv, Connected Papers, and Semantic Scholar. These platforms were chosen to cover both peer-reviewed and preprint literature, and to represent diverse indexing strategies across social sciences, public health, and computer science. The search was performed in multiple rounds from April to July 2024, with backward and forward citation chaining to ensure thematic saturation.

The chosen time frame spans from January 1, 2012, to July 1, 2024. This range was selected to capture the rise of deep learning in NLP as well as key public health moments (notably the COVID-19 pandemic) that significantly influenced both media coverage and computational research trends in mental health.

**Fig. 2:**
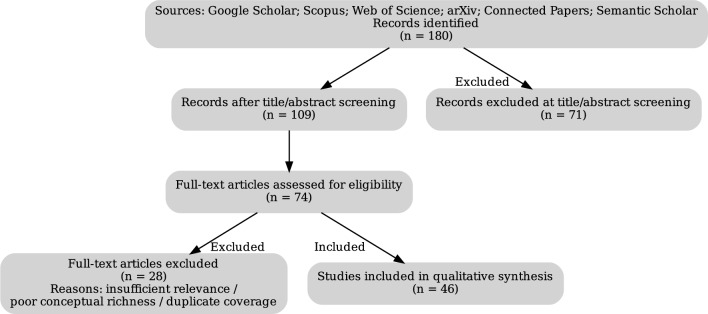
Flow diagram of the scoping review. Records were identified (n=180) after title/abstract screening (n=109), full-text assessment (n=74), full-text excluded (n=28), and studies included (n=46).

Our initial search yielded approximately 180 documents (see Fig. 2). We screened titles and abstracts for relevance to both (a) the application of NLP methods and (b) the context of mental health in news or social media. Publications focused solely on clinical health records, without a media component, or that employed NLP in unrelated contexts (e.g., biomedical entity extraction) were excluded. After full-text review, 46 publications were selected based on conceptual richness, relevance to the intersection of news media and mental health, and the diversity of NLP techniques applied.

To contextualize our approach, we emphasize that this study was designed as a scoping review rather than a systematic review. Our review process follows principles of scoping reviews, designed to capture a broad and interdisciplinary sample rather than apply a narrowly systematic protocol. By including diverse sources across public health, computer science, and social sciences, we aimed to map methodological variety and highlight key trends and gaps. While not exhaustive, the final set of 46 studies provides a sufficiently representative overview to derive meaningful insights and recommendations.

In this review, we define “news media” as any form of digitally available textual publication by news broadcasting services. This includes information-based texts that report on events and occurrences, as opposed to social media texts that primarily share opinions and positions. News media texts differ from social media texts in that the latter use informal, direct language with slang and abbreviations^22^.

During our analysis of the different NLP techniques, our attention was directed towards three primary factors. These were chosen to reflect not only the technical capacities of NLP tools, but also their relevance to understanding and improving mental health representations in public discourse. First, we assessed how NLP techniques can detect complex psychological constructs (such as depression, anxiety, and well-being) in media narratives. Second, we examined the precision and depth of analysis these tools offer, emphasizing the portrayal of mental health in a balanced and accurate manner. Third and last, we identified key gaps in existing research and suggested areas for further development to enhance the role of NLP in improving mental health portrayals.

By “key gaps”, we refer to recurring methodological or ethical limitations observed across the reviewed literature. These include the dominance of English-language datasets and tools, limited model transparency and explainability, insufficient cultural or contextual adaptation, and the lack of interdisciplinary evaluation frameworks tailored to mental health media analysis.

### Traditional content analysis in psychology and social sciences

Traditional approaches to content analysis have been instrumental in the study of media representations and their impact on public perceptions in psychology and the social sciences. While these methods are labor-intensive and limited in scope, they provide important insights into how mental health is portrayed in the media. The report depends on the type of content analysis, whether qualitative or quantitative.

Qualitative content analysis is a method that involves the systematic and subjective interpretation of textual data through the classification of themes or patterns. Central to this approach is the development of specialized lexicons for specific constructs related to mental health, such as depression or anxiety. Manual coding by experts, guided by predefined coding rules and decision trees, ensures detailed and contextual analysis. This method captures subtle implications and contexts that automated methods may miss. However, it is labor-intensive, subjective, and not scalable to large datasets^23^.

Quantitative content analysis systematically quantifies the presence of specific words, phrases, or themes in a text using statistical techniques. Coding schemes define categories for analysis, and the data is statistically analyzed to identify patterns and trends. This method offers greater objectivity and efficiency, allowing for the processing of larger data sets. However, it can miss nuanced meanings and contextual subtleties within the text, limiting its ability to capture the full complexity of media representations^24^.

Researchers often use mixed methods, integrating both techniques for a comprehensive analysis to leverage the strengths of both qualitative and quantitative methods. This iterative process allows for a nuanced understanding, followed by the validation and generalization of findings. While providing balanced insights, combined approaches are complex and resource-intensive, requiring expertise in both qualitative and quantitative techniques^25,26^.

### NLP for news media and mental health

**Fig. 3:**
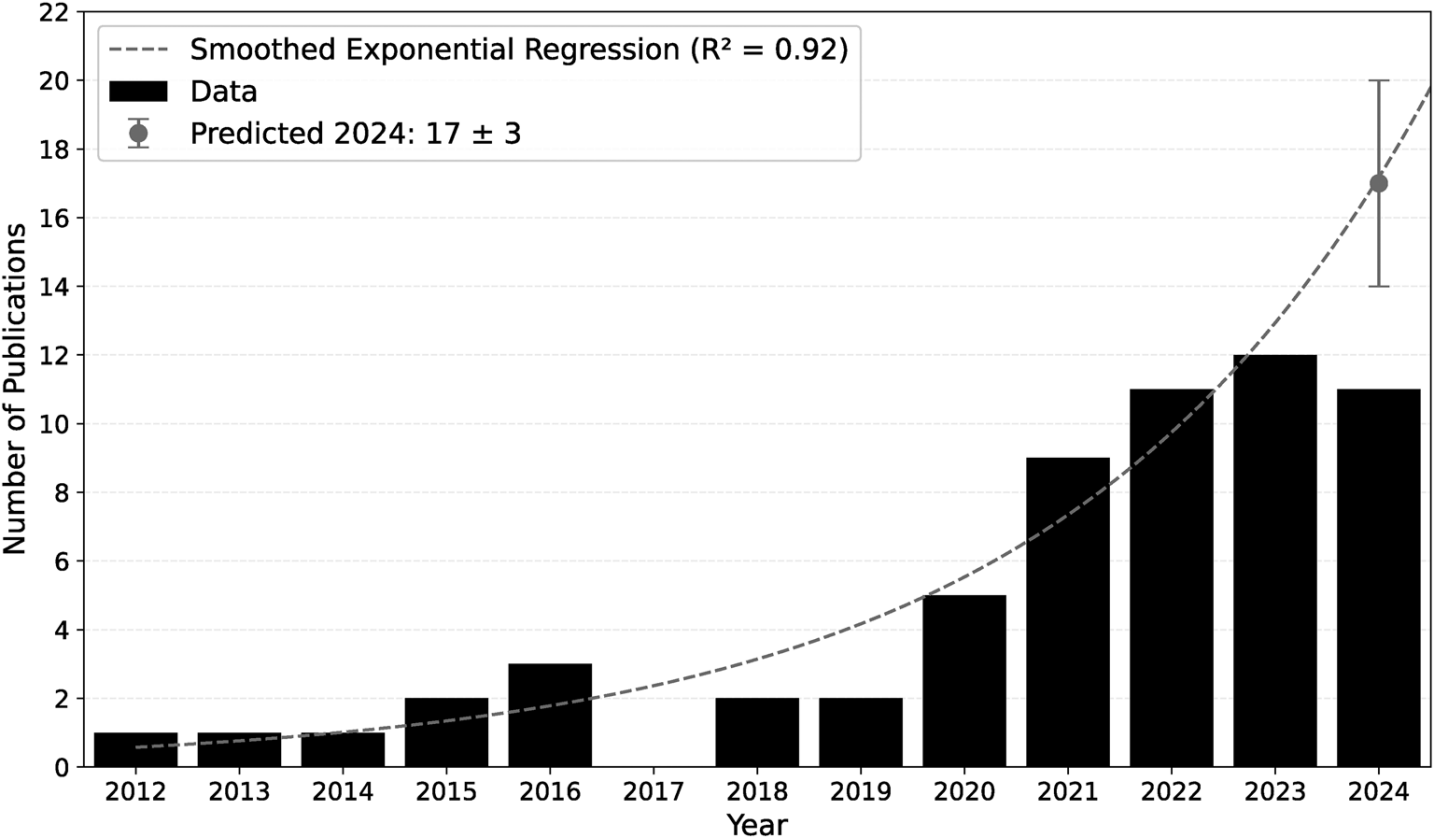
Yearly Distribution of Publications. The figure illustrates the number of reviewed and published publications on applying natural language processing in news media and mental health from 2012 to 2024. An exponential regression Line, fitted to the publication data, indicates an upward trend in research activity. The prediction for 2024 suggests continued growth in this research area.

Examining the trends in NLP for news media and mental health analysis reveals a significant increase in research activity, particularly around 2021 and 2022. This trend is expected to continue, driven by several factors; Interest in understanding the impact of the media on public well-being has grown with increased awareness of mental health. Advances in NLP technology, particularly with models like Bidirectional Encoder Representations from Transformers (BERT)^27^ and Generative Pre-trained Transformer (GPT)^28^, have enabled more sophisticated text analysis. Additionally, there is growing interest in digital health solutions, such as mental health monitoring through media analysis. Ethical and inclusive development in NLP also drives research to ensure that AI tools responsibly serve diverse populations^29^. These factors collectively fuel the expansion of this field (see Fig. 3).

**Fig. 4:**
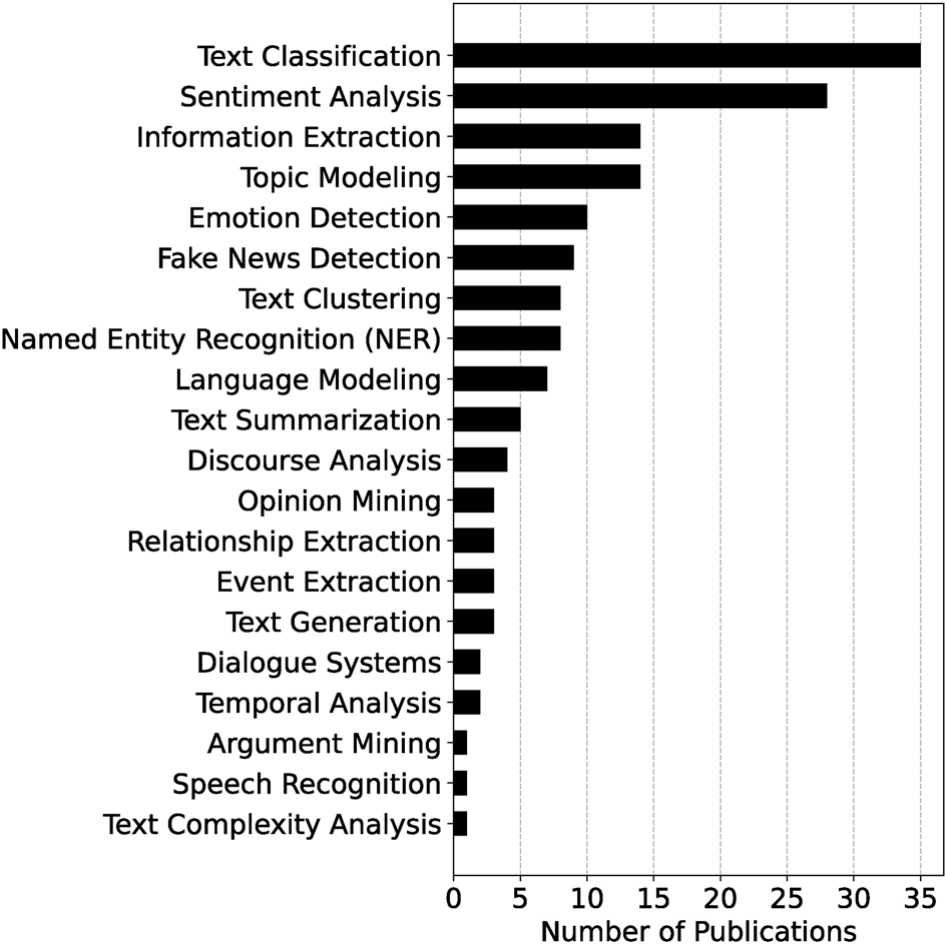
Number of publications by Natural Language Processing Task for News Media and Mental Health. This graph illustrates the distribution of research publications across different NLP tasks. The data is sorted in ascending order based on the number of studies. Notably, ‘text classification’ and ‘sentiment analysis’ are the most relevant tasks in the field, reflecting their critical importance and wide applicability in the NLP domain. On the other hand, tasks such as ’Suicidal Tendency Prediction’ and ’Speech Recognition’ have less related work, indicating potential areas for future research focus.

We illustrate how advances in NLP are critical to addressing the challenges of analyzing mental health narratives in the news media by examining techniques used in the field. NLP tasks such as sentiment analysis and topic modeling have proven particularly valuable in identifying patterns in media representations of mental health. Sentiment analysis, for example, helps measure the emotional tone of news articles or phrases, revealing potential biases in coverage that contribute to stigma or misunderstanding. Topic modeling uncovers recurring themes, such as the association of mental illness with crime or social instability, that shape public perceptions. Other NLP tasks, such as text classification, have also been explored in the context of mental health and the media. However, much of the research remains focused on detecting misinformation rather than mental health narratives specifically. As shown in Figure 4, sentiment analysis and text classification are among the most commonly used techniques in news media-related publications, reflecting their critical role in understanding the framing of mental health issues.

Recent advances in deep learning have significantly improved natural language understanding (NLU) capabilities, particularly in media analysis. Transformer-based architectures have been particularly effective, outperforming earlier models such as Recurrent Neural Networks (RNNs) and Long Short-Term Memory (LSTM) networks^30–32^ in capturing the nuances of mental health narratives in news media. Large language models (LLMs), built on transformer architectures, have improved the ability to analyze mental health-related content in news articles. These models excel at capturing long-range dependencies and complex linguistic patterns, which are critical for understanding nuanced language in mental health contexts. For example, the BERT model achieved cutting-edge performance on the General Language Understanding Evaluation (GLUE) benchmark with a score of 80.5%^27^, demonstrating its superior language understanding capabilities. In mental health-specific tasks, domain-adapted models have shown remarkable performance. MentalBERT and MentalRoBERTa, introduced by Ji et al.^33^, outperformed general-purpose models in detecting mental disorders from social media text. On the eRisk 2018 depression detection task, MentalBERT achieved an F1 score of 0.86, compared to 0.83 for BERT-base. Garg^34^ developed the WELLXPLAIN dataset for wellness dimension classification and compared different models, including GPT-3 and MentalBERT. Their study showed that GPT-3 achieved the highest accuracy at classifying wellness dimensions at 72.3%, followed closely by MentalBERT at 71.8%. While true NLU remains a complex goal that requires consideration of philosophical, cognitive-linguistic, and technical perspectives, transformers have become the preferred basis for many NLU tasks. They consistently outperform RNN-based models on various benchmarks, including those relevant to mental health analysis in media contexts.

LLMs extend media content analysis capabilities by providing scalable NLP solutions. They excel at tasks ranging from text generation to summarization and entity recognition, helping to decipher mental health narratives in media. However, issues of interpretability and hallucination risk require caution in critical domains such as mental health. While most related work focuses on single-modal data, multimodal approaches that integrate text, audio, and images are gaining interest^35^. These methods, while less common, promise a more comprehensive view of mental health representations by considering both verbal and nonverbal cues in media such as news broadcasts and social media posts. This area remains largely unexplored and offers opportunities for future research.

#### NLP for news media

NLP has become an indispensable tool for analyzing the vast amounts of textual content generated by news outlets. It allows researchers to explore the structure, framing, and bias of media coverage, providing valuable insights into how public perception is shaped. Using methods such as text classification, topic modeling, and sentiment analysis, NLP enables the detection of misinformation and media framing, providing a more nuanced understanding of how news is communicated and its impact on society.

Traditional media use formal language and adhere to professional journalistic standards to provide objective, balanced information. In contrast to the informal, subjective content typical of social media, traditional news articles are structured, detailed, and comprehensive, contributing significantly to opinion formation and narrative delivery. This includes a variety of reporting formats, such as news articles, editorials, and commentaries. Understanding these linguistic and structural characteristics is critical to applying NLP techniques to news content, particularly when analyzing how mental health issues are framed.

Related work in this area has demonstrated the utility of NLP for analyzing media bias^36–38^, extracting semantic knowledge^36^, and understanding political framing^39,40^. For example, NLP has been instrumental in tracking COVID-19 news coverage^41^, assessing the accuracy of information shared, and identifying linguistic patterns in reporting. In public health contexts, NLP has enabled event-based monitoring of news articles^42^, revealing patterns of misinformation^43^ or bias that influence health-related behaviors and attitudes. The structured and professional nature of traditional news media provides a well-defined linguistic framework for NLP applications, allowing for more accurate detection of bias, framing, and thematic patterns. However, while much progress has been made, these tools still focus primarily on topics such as political reporting or general public health, with less emphasis on mental health-specific topics.

#### NLP for mental health

NLP for Mental Health has gained traction as a transformative tool for analyzing linguistic patterns associated with mental health conditions. NLP techniques are used to identify emotional states, assess mental health risks, and personalize interventions based on language use. Recent publications have explored the potential of NLP for emotion recognition, identification of at-risk individuals, and personalization of mental health support^15,44^. For example, NLP techniques have been used to analyze text-based communication in crisis support services to identify linguistic cues that indicate heightened emotional distress^45^, and to assess communication patterns in counseling^46^, helping to refine intervention strategies. Text classification in this domain can be used to categorize individuals’ mental states based on their language use, while emotion detection can identify specific emotional shifts in patients, contributing to more effective interventions and treatment plans.

In clinical settings, NLP models have demonstrated potential for extracting psychopathological cues from text, analyzing therapy transcripts, and providing computational support for traditional diagnostic methods^47^. For example, the analysis of language in patients with bipolar disorder^48^ and other mental illnesses^49,50^ has shown promising applications for early diagnosis, treatment evaluation, and ongoing mental health monitoring. NLP-based systems can effectively detect depression severity from social media posts, aligning with clinical metrics such as PHQ-9 and underscoring the importance of emotion detection and text classification in mental health interventions^51^. Similarly, in clinical settings, transformer models such as Bio_ClinicalBERT have outperformed traditional methods in extracting personal and family histories of suicidal behavior from EHRs, aiding in risk assessment^52^.

Large language models applied to Reddit posts have revealed topics related to suicidality, consistent with modern suicide theories, by identifying common patterns of distress^53^. Beyond diagnostics, AI-driven NLP tools extend into patient engagement, with personalized chatbots improving mental health service access, increasing referrals, and reducing stigma^54^.

However, significant challenges remain. Issues such as language bias, ethical use of data, and concerns about data quality are major obstacles^55^. As the field continues to evolve, more research is needed to address these limitations and refine the applicability of NLP models in mental health interventions^56^.

The application of NLP to news media on mental health enables the systematic analysis of how mental illness is portrayed and how such portrayals may shape public perception and stigma. Given the media’s influence on societal attitudes and health behaviors, this intersection is a key focus for advancing public mental health through informed and balanced reporting.

Sentiment analysis has been applied to mental health news coverage, uncovering the emotional biases that influence public understanding of mental health disorders. For example, media outlets might frame mental illness negatively, associating it with violence or danger, which can reinforce public stigma. Topic modeling in this intersection has helped identify recurring themes in media coverage of mental health^57,58^, such as the association between mental illness and societal instability, which may perpetuate negative stereotypes. By analyzing the frequency and tone of these topics, NLP can provide insights into how mental health is discussed across different news outlets, shedding light on societal attitudes towards mental health issues^12,13^. In addition, NER can be used to track specific entities (such as organizations, public figures, or mental health conditions) and how they are discussed in relation to mental health in the media. This can highlight patterns of misinformation or under-reporting of critical mental health issues and influence public understanding and policy development. It has already been successfully applied to extract psychiatric attributes from German mental health records^59^, using GermanBERT^60^. Recent work has demonstrated tangible progress in extending NLP capabilities to low-resource settings. For instance, Ronny Mabokela et al.^61^ developed sentiment classifiers for five Southern African languages–Sepedi, Setswana, Sesotho, isiZulu, and isiXhosa–using multilingual pretrained language models such as AfroLM and mBERT, achieving strong F1 scores through ensemble learning and language-specific tuning. Similarly, Joshi et al.^62^ introduced Nemotron-Mini-Hindi, a bilingual Hindi-English language model adapted through continued pretraining on synthetic corpora, which significantly improved benchmark performance on Indic tasks. These studies highlight scalable, transferable techniques that can address linguistic inequality in NLP research.

The intersection of NLP in news media and mental health opens up new possibilities for analyzing how mental health issues are framed and perceived by the media. By applying techniques such as sentiment analysis, topic modeling, and misinformation detection, researchers can better understand how news outlets shape public discourse about mental health and how this framing influences societal attitudes and stigma. While these advances are promising, a review of the existing literature reveals several gaps and challenges that must be addressed to fully realize the potential of NLP in this area. These gaps include issues such as language bias, limited data diversity, and a lack of interpretability in LLMs. Addressing these challenges requires targeted recommendations for NLP developers and researchers working at the intersection of mental health and news media.

#### Recommendations

The related work provides valuable insights into developing and performing models tailored to detect stress, bias, and mental disorders in social media and other text data. It highlights the effectiveness of domain-specific embeddings, the importance of extensive context for long-sequence modeling, and the need for bias-neutralizing tools. However, they also point out significant limitations, such as the reliance on English-language data, potential biases in training data, and the challenges in handling longer text sequences. These insights form the foundation for the following ten recommendations, which aim to address the identified gaps and propel future research and applications toward more robust, fair, and comprehensive NLP solutions in mental health and news media analysis:

1. It is essential to develop and utilize comprehensive datasets that are representative of the diversity found in news media content. This should include different languages, regions, and media types to ensure models are generalizable across various contexts. Domain-specific datasets from social media and medical health records are also crucial for effectively fine-tuning language models such as Ji et al.^33^.
2. Strategies must be implemented to detect and mitigate biases in both training data and models. Addressing bias is critical to achieving objective and fair analysis outcomes in mental health and news media NLP applications.
3. Rigorous validation and testing of NLP models should be performed to ensure their reliability and accuracy. Models must be tested against diverse real-world scenarios to ensure robustness and applicability.
4. Pre-trained domain-specific models such as MentalBERT and ClinicalBERT should be leveraged to enhance the relevance and accuracy of analyses, particularly in mental health. Despite their utility, the current lack of explainability in LLMs requires ongoing expert validation of their results.
5. Multi-dimensional analysis pipelines should be developed. These pipelines should incorporate sentiment analysis tailored to mental health discourse, topic modeling for emerging issues, and NER to identify mental health resources and events.
6. Improving the interpretability of LLMs is crucial. Techniques should be developed to make these models more transparent and understandable, facilitating a clearer understanding of their decision-making processes.
7. Greater attention should be given to linguistic diversity. NLP techniques must be designed to handle a broad range of languages and dialects, addressing the current bias toward English-language data and supporting the development of multilingual language models.
8. Improving reproducibility in NLP research is vital. Standardizing evaluation metrics and enhancing the availability of open data and code will contribute to greater transparency and replicability in the field.
9. Focusing on multimodal analysis that incorporates text, images, and audio is recommended to provide richer and more accurate insights into mental health and media representation.
10. An emphasis on ethical AI practices is essential. NLP applications in mental health and news media must be developed with fairness, transparency, and responsibility in mind to ensure they serve the broader public good.

These recommendations serve as a guide for NLP developers aiming to work at the intersection of news media and mental health. They focus on improving the quality, fairness, and transparency of NLP applications.

## NLP techniques

Building on the challenges and recommendations, the next sections provide a detailed summary of the NLP techniques used in the reviewed related work. By categorizing these techniques by task, we aim to provide domain experts with a clear and organized overview of the approaches most applicable to specific problems in news media and mental health analysis.

### Discovering news media content through multimodal deep learning techniques

Somandepalli et al.^63^ used multimodal learning to analyze media representations of gender, race, and age. They integrated audio, video, and text data using NER, sentiment analysis, and RNNs with attention mechanisms. Their approach included a mixture-of-experts model for predicting emotional responses and cross-modal autoencoders for classifying TV commercials. This methodology offers potential for systematic analysis of media narratives, particularly in the portrayal of mental health. Zhang^64^ presented DCLSTM-MLP, a news text classification model that combines Convolutional Neural Network (CNN), LSTM, and MLP. It uses word vectors and word scatter to capture spatiotemporal relationships and word-category associations. Experiments demonstrated DCLSTM-MLP’s superior performance in accuracy, recall, and overall measures compared to existing methods, thus advancing automatic news classification in NLP.

### Topic modeling

Analysis of Topic Model Networks (ANTMN), introduced by Walter and Ophir^65^, uses Latent Dirichlet Allocation (LDA)^66^ for topic modeling, network analysis, and community detection to identify media frames. It constructs networks with topics as nodes and cosine similarity as edges, using algorithms such as Walktrap^67^ and Louvain^68^ for frame clustering. Ghasiya and Okamura^69^ analyzed COVID-19 news using Top2Vec^70^ and Robustly optimized BERT approach (RoBERTa)^71^ for sentiment analysis, revealing cross-cultural differences in media coverage and public sentiment. Choi and Um^72^ used LDA and Structural Topic Modeling (STM) to analyze Korean COVID-19 news, identifying five major themes and highlighting the underrepresentation of psychological effects in media coverage. Lu et al.^73^ used Sentence-Bidirectional Encoder Representations from Transformers (Sentence-BERT)^74^, spaCy^75^, and Top2Vec to analyze economic news during COVID-19, revealing shifts in media focus from health to economic impacts over time. The related work demonstrates the effectiveness of combining topic modeling and sentiment analysis in understanding media narratives and public responses during global crises and highlights the influence of cultural and political contexts on media coverage.

### Sentiment analysis

Nemes and Kiss^76^ analyzed COVID-19 tweets using BERT, RNNs, and Natural Language Toolkit (NLTK) with VADER^77^. They combined information extraction (IE), NER, and sentiment analysis for contextual understanding. Succar et al.^78^ used DeBERTa^79^ for aspect-based sentiment analysis of Twitter data, using transfer entropy and convergent cross mapping (CCM) to assess media-sentiment relationships. Gottipati et al.^80^ studied media representations of mental disorders using NLP and sentiment analysis. Lin et al.^81^ developed a BERT-based ensemble model to identify harmful news content. Mittal and De Choudhury^82^ compared moral framing in mental health discourse between social media and news using a BERT-based framework, demonstrating that Twitter is more aligned with positive moral foundations compared to news articles. The related work showcases advanced NLP techniques for sentiment analysis in different contexts, highlighting their potential for understanding public discourse and media impact on sensitive issues.

### Identifying bias and framing in news media

Spinde et al.^37^ developed a bias detection method for German news using Inverse Document Frequency (IDF), bias lexicons, and word embeddings. Acken and Demszky^39^ analyzed the framing of the 2020 presidential election using Named Entity Recognition (NER) and semantic lexicons^83^. Lei et al.^38^ used the Robustly Optimized BERT Approach (RoBERTa) and BiLSTM models with knowledge distillation^84^ for sentence-level bias analysis. Choubey et al.^85^ proposed an Integer Linear Programming (ILP) system for event coreference resolution using discourse structures. Doumit and Minai^36^ combined Latent Dirichlet Allocation (LDA) and Antelope^86^ to analyze media bias through cognitive networks. Bach et al.^40^ used fine-tuned BERT embeddings to analyze political news consumption in three European countries, linking browsing patterns to political engagement. These approaches demonstrate advanced NLP techniques for detecting bias, framing, and content structure in news media and provide scalable methods for political communication research and media analysis.

### Fake news detection

Kaliyar et al.^20^ proposed FakeBERT, which combines BERT with CNNs for improved fake news detection, achieving 98.90% accuracy on a U.S. presidential election dataset. Nasir et al.^21^ presented a hybrid CNN-RNN model evaluated on FA-KES^87^and ISOT datasets, highlighting the importance of generalization techniques^88^. presents a hybrid approach combining NLP and expert opinion, using BERT-based architectures and blockchain technology for transparent news authenticity assessment. The related work demonstrates the evolution of fake news detection techniques, from pure NLP approaches to hybrid models that integrate human expertise and advanced machine learning architectures.

### Mental health assessment from text

Murarka et al.^50^ used the Robustly Optimized BERT Approach (RoBERTa) to classify mental health posts on Reddit, outperforming the LSTM and BERT models. Ji et al.^89^ reviewed methods for identifying suicidal ideation, highlighting CNNs, LSTMs, and hybrid models such as BERT with Gated Recurrent Units (GRU). Garg^34^ developed the WELLXPLAIN dataset for wellness dimension classification, comparing various models including Generative Pre-trained Transformer 3 (GPT-3) and MentalBERT. Turcan and McKeown^90^ presented “Dreaddit” to identify stress in social media using logistic regression and neural models. Ji et al.^91^ developed MentalXLNet and MentalLongformer for long-sequence modeling in mental health, outperforming BERT and RoBERTa in certain tasks. Choey^92^ created a system to neutralize biased language in discussions of mental illness, based on the CONCURRENT model^93^. Ji et al.^33^ presented MentalBERT and MentalRoBERTa, pre-trained models for detecting mental disorders from social media text. The related work demonstrates advanced NLP applications in mental health assessment and highlights the potential and limitations of different models and datasets.

### Large language models (LLMs)

LLMs have significantly advanced the analysis of mental health narratives by improving existing NLP methods. The related work demonstrates the potential of LLMs for detecting mental health disorders and analyzing social media data^53,94,95^. LLMs show promise in psychological assessment^96^ and analysis of online depressive and suicidal behavior^97^. Fine-tuned models such as MentalBERT, MentalRoBERTa^33^, MentalXLNet, and MentalLongformer^91^ provide nuanced text understanding. However, concerns remain about bias in artificial intelligence (AI) models of mental health^98^. Rigorous evaluation of LLMs against traditional methods is critical to validate their efficacy and reliability. Researchers emphasize the need for collaboration between clinicians and data scientists to address biases and ensure equitable mental health care^99^.

## Discussion

The review paper provides an in-depth analysis of various NLP techniques used for news media analysis, with a specific focus on mental health-related issues. The introduction highlighted the importance of understanding how mental health is represented in the media and the potential impact on public perception and stigma. The methods section explored various NLP techniques, including sentiment analysis, topic modeling, and fake news detection, and examined their applicability, benefits, and limitations in analyzing news media content. The application of NLP in news media and mental health includes various methods, each of which has its strengths and limitations:

First, various NLP techniques are very effective at processing large amounts of news media content at scale, providing automated analysis that traditional manual methods cannot achieve. This scalability allows for continuous monitoring of media representations, making it possible to identify emerging trends in discussions of mental health. In addition, this automated approach enables real-time monitoring and rapid identification of negative portrayals of mental health, facilitating timely intervention. Public health officials and policymakers can use these insights to promptly address stigmatizing content or misinformation and promote more supportive narratives.

Second, sentiment analysis in NLP helps track public sentiment about mental health over time, providing valuable insights into societal reactions to media coverage. Combined with its real-time capability, sentiment analysis is particularly useful for monitoring the impact of news stories on public perception and mental health stigma.

Third, topic modeling is another key strength of NLP for this domain. It allows the identification of dominant topics in news media. For example, it has been used to analyze the portrayal of mental health during crises such as COVID-19^57,72,73^, helping researchers uncover how different narratives are framed across regions and time periods. It is worth noting here that topic modeling lacks robust benchmarking tests, making it difficult to assess its consistency and reliability across datasets, largely due to its unsupervised nature and variability in results.

Fourth, NLP’s ability to detect fake news and misinformation using advanced models such as FakeBERT^20^ and hybrid CNN-RNN architectures^21^ enhances the credibility of media content. This is critical to ensuring that mental health is accurately represented in the news and minimizing the spread of harmful misinformation.

Fifth, a key benefit of NLP is its ability to provide objective and consistent analysis by minimizing the influence of personal bias. Using machine learning models, NLP reduces the subjectivity that often accompanies manual content analysis, leading to more reliable and standardized results when assessing media portrayals of mental health. However, one must still account for bias that may be inherent in the data itself or introduced into the analysis in hidden ways by LLMs.

Sixth, despite these strengths, a major limitation of NLP lies in the interpretability of LLMs, which often function as “black boxes”. Their decision-making processes are opaque, making it difficult to validate results or ensure the accuracy of predictions, especially when dealing with sensitive topics such as mental health.

Seventh, bias in training data is another significant challenge. NLP models trained on biased datasets run the risk of perpetuating biases in their analysis, potentially reinforcing harmful stereotypes about mental health. Addressing this issue is critical to ensure fair and unbiased media analysis.

Eighth, NLP’s overreliance on English-language data limits its ability to provide comprehensive global insights. Current models have an English bias, which limits their applicability to diverse linguistic contexts. The development of multilingual NLP systems is critical to accurately analyze mental health representations across cultures and languages. The success of ensemble-based PLMs in African languages^61^ and the continued pretraining of bilingual LLMs^62^ offer promising strategies to advance NLP inclusivity across both formal and informal media in low-resource contexts.

Ninth, while NLP excels at large-scale automated analysis, it often fails to capture the rich contextual depth provided by traditional qualitative methods. This limitation highlights the need to combine NLP with manual approaches to gain a more nuanced understanding of mental health representations in the media^45^.

Tenth, the issues of reproducibility and transparency also challenge the efficacy of NLP, which aligns with the findings of Malgaroli et al.^15^. The lack of standardized metrics, coupled with the limited availability of open data and code, hinders the ability to replicate previous published work and validate findings, which is critical for reliable research on mental health representations in the media.

### Major findings of this review

Before turning to future research directions, we summarize here the major findings that emerge from the reviewed literature:

- **NLP enables large-scale analysis of mental health discourse:** Automated methods make it possible to study news media coverage at scales far beyond manual content analysis, revealing long-term patterns and trends (see Section Introduction, Sect. Discussion).
- **Emotional framing and stigma can be systematically measured:** Sentiment analysis has been used to capture emotional framings of mental health, showing that media often convey negative or fear-related associations (see Section Sentiment Analysis).
- **Topic modeling highlights dominant themes but lacks benchmarking:** Studies have identified recurring topics such as depression, suicide, and public health crises, yet systematic benchmarking across methods and corpora remains limited (see Section Topic Modeling).
- **Misinformation and credibility assessment are emerging areas:** NLP has been applied to detect misinformation in mental health reporting, though applications remain sparse and exploratory (see Section Fake News Detection).
- **Bias, language coverage, and interpretability remain key challenges:** Current work is restricted largely to English corpora, with limited attention to transparency or fairness. Data biases risk reinforcing stereotypes, and the interpretability of large language models remains a pressing issue (see Section Overview of NLP Applications in Mental Health and News Media).

Together, these findings demonstrate both the promise of NLP for understanding mental health discourse in news media and the critical gaps that future work must address.

### Future research and development

Future research in NLP for news media analysis should prioritize four key areas to improve the effectiveness and fairness of these methods.

First, improving the interpretability and transparency of large language models (LLMs) is critical. Developing strategies that make these models more explicable will help in understanding their decision-making processes. In addition, combining qualitative and quantitative approaches can leverage the strengths of both methods to provide a more comprehensive analysis. Addressing biases in training data and models is essential to achieving objective results, especially since many current systems are biased toward English data and lack linguistic diversity. Advancing multilingual NLP capabilities will allow better analysis of global news media and promote more inclusive research efforts.

Second, ethical considerations must be at the forefront of NLP research. Emphasizing ethical AI practices will ensure that applications are fair and responsible. In particular, unintended consequences of automated classification–such as the mislabeling of sensitive or stigmatizing content–pose ethical risks. These require mechanisms for uncertainty estimation, human-in-the-loop validation, and continuous retraining. Moreover, interpretability is paramount: real-world deployment of NLP in high-stakes contexts like mental health should be accompanied by model explanation tools and regular expert review. Developing explainable AI techniques will increase the trustworthiness and usability of NLP models. Continuous integration of expert evaluation into the development and validation processes is essential to maintain accuracy and reliability. In addition, addressing privacy and security concerns is critical; researchers should develop methods for anonymizing data without sacrificing analytical value to protect sensitive information.

Third, practical applications of NLP should include the creation of real-time analytics systems that can identify and flag harmful or stigmatizing content in a timely manner. Such systems would facilitate faster responses and promote supportive narratives in media coverage. It is also important to develop user-friendly tools that are accessible to a wide range of users, including journalists and policymakers. Training programs can be established to help users make effective use of these tools. In addition, it is essential to develop comprehensive evaluation frameworks to assess the impact of NLP tools on media portrayals and public perceptions of mental health, including both quantitative and qualitative measures.

Fourth and finally, exploring the broader societal and policy implications of NLP-based media analysis is critical. Research should focus on developing strategies that use insights from NLP analysis to inform public health campaigns and policy decisions that promote accurate representations of mental health in the media. Interdisciplinary collaboration between NLP researchers, mental health professionals, and media experts will be essential to developing well-rounded approaches. By addressing these key areas, future research can significantly improve the effectiveness, fairness, and applicability of NLP techniques in the analysis of news media content related to mental health. This version includes transitional phrases to clearly delineate each point.

## Conclusion

This review set out to examine how NLP techniques can help identify, interpret, and improve media portrayals of mental health. We surveyed both traditional and advanced methods–such as topic modeling, contextual embeddings, and emotion detection–and assessed their capacity to capture nuanced representations of psychological constructs in the news. We highlighted key limitations, including language bias, lack of explainability, and insufficient attention to stigmatizing versus supportive framing. To address these challenges, we proposed ten actionable recommendations aimed at guiding the development of more accurate, ethical, and socially aware NLP tools. Looking ahead, interdisciplinary collaboration will be crucial to fully realizing the potential of NLP in promoting more balanced and empathetic mental health discourse across media platforms.

## Data Availability

The datasets generated during and/or analysed during the current study are available from the corresponding author on reasonable request.
